# Protective role of the dynamin inhibitor Dynasore against the cholesterol-dependent cytolysin of *Trueperella pyogenes*

**DOI:** 10.1096/fj.14-265207

**Published:** 2014-12-30

**Authors:** Giulio Preta, Virginia Lotti, James G. Cronin, I. Martin Sheldon

**Affiliations:** Institute of Life Science, College of Medicine, Swansea University, Swansea, United Kingdom

**Keywords:** *host-pathogen interaction*, *bacteria*, *mammalian cell survival*, *lipid rafts*

## Abstract

The virulence of many Gram-positive bacteria depends on cholesterol-dependent cytolysins (CDCs), which form pores in eukaryotic cell plasma membranes. Pyolysin (PLO) from *Trueperella pyogenes* provided a unique opportunity to explore cellular responses to CDCs because it does not require thiol activation. Sublytic concentrations of PLO stimulated phosphorylation of MAPK ERK and p38 in primary stromal cells, and induced autophagy as determined by protein light-chain 3B cleavage. Although, inhibitors of MAPK or autophagy did not affect PLO-induced cytolysis. However, 10 *μ*M 3-hydroxynaphthalene-2-carboxylic acid-(3,4-dihydroxybenzylidene)-hydrazide (Dynasore), a dynamin guanosine 5′-triphosphatase inhibitor, protected stromal cells against PLO-induced cytolysis as determined by 3-(4,5-dimethylthiazol-2-yl)-2,5-diphenyltetrazolium bromide assay (85 ± 17% *versus* 50 ± 9% cell viability), measuring extracellular ATP, and kinetic assays. This was a generalized mechanism because Dynasore also protected HeLa cells against streptolysin O. Furthermore, the effect was reversible, with stromal cell sensitivity to PLO restored within 30 minutes of Dynasore removal. The protective effect of Dynasore was not conferred by dynamin inhibition, induction of ERK phosphorylation, or Dynasore binding to PLO. Rather, Dynasore reduced cellular cholesterol and disrupted plasma membrane lipid rafts, similar to positive control methyl-*β*-cyclodextrin. Dynasore is a tractable tool to explore the complexity of cholesterol homeostasis in eukaryotic cells and to develop strategies to counter CDCs.—Preta, G., Lotti, V., Cronin, J. G., and Sheldon, I. M. Protective role of the dynamin inhibitor Dynasore against the cholesterol-dependent cytolysin of *Trueperella pyogenes*.

Bacteria have developed many strategies that impact the cellular functions of the host; the pore-forming toxins constitute the largest class of bacterial virulence factors, comprising approximately 25% of all bacterial protein toxins (1, 2). The mechanism of action of pore-forming toxins is simple yet elegant because they form holes in the plasma membrane, disrupting membrane integrity and ion homeostasis (3, 4). Cholesterol-dependent cytolysins (CDCs) are a large family of *β*-barrel pore-forming toxins that are produced by Gram-positive bacteria (5, 6). Pore formation is dependent on the presence of membrane cholesterol, which functions as the receptor for most CDCs. Cholesterol binding initiates significant secondary and tertiary structural changes in the CDC monomers, which leads to the assembly of a large membrane-embedded *β*-barrel pore complex. Interestingly, disparate cell types treated with different CDCs can display similar physiologic responses, including increase in cytosolic calcium, conformational changes, and cytoplasmic vacuolization (7). The functional significance of these responses as well as the responses at the molecular level is poorly understood.

Among the less-characterized CDCs, pyolysin (PLO) is the most important actively secreted virulence factor of *Trueperella pyogenes*, which is an opportunistic pathogen causing mastitis and endometritis in ruminants (8). After parturition, purulent disease of the endometrium is evident in up to 40% of dairy cows (9). This infection reduces milk production and causes infertility, costing U.S. and European dairy industries $2 billion annually (9). Exposure to high concentrations of CDCs leads to cell death, which can be either apoptotic or necrotic (10). However, during infection, *in vivo* host cells are initially exposed to sublytic concentrations of CDCs, which may allow the cells time to respond to the insult. In the present study, the aim was to examine the responses induced by sublytic concentrations of PLO, in regard to 3 major pathways: 1) MAPK, 2) autophagy, and 3) cellular cholesterol. The MAPK family is a group of highly conserved protein-serine/threonine kinases, involved in intracellular regulation in response to various stresses. The MAPK p38, JNK, and ERK1/2 are activated as a defense response by eukaryotic cells to pore-forming toxins (3, 11, 12). Autophagy is also activated in response to pore-forming toxins probably to maintain energy supply as cells enter a quiescent state upon pore formation, while plasma membranes are repaired (3, 13, 14). Finally, cholesterol content and intracellular cholesterol trafficking are important for responses to CDCs because modification of the levels of membrane cholesterol affects pore formation and the sensitivity of host cells (15).

In the present study, treatment of primary endometrial stromal cells with sublytic concentrations of PLO induced phosphorylation of MAPK and autophagy. However, inhibitors targeting MAPK or autophagy pathways provided minimal protection for cells against PLO. Inhibitors that conferred durable protection against PLO were the dynamin guanosine 5′-triphosphatase (GTPase) inhibitor 3-hydroxynaphthalene-2-carboxylic acid-(3,4-dihydroxybenzylidene)-hydrazide (Dynasore), and the cyclodextrin methyl-*β-*cyclodextrin (M*β*CD). We previously showed that cyclodextrins reduce endometrial stromal cell sensitivity to PLO by decreasing cellular cholesterol content (15), whereas Dynasore impairs cholesterol trafficking and sterol-sensitive gene transcription in human HeLa cells and macrophages (16, 17). Moreover, Dynasore inhibits the entry of several viruses, intracellular bacteria, and parasites (18–20). In our study, Dynasore not only protected bovine endometrial stromal cells against PLO but was also effective with HeLa cells and streptolysin O (SLO). We provide evidence that Dynasore acts mainly by targeting lipid rafts and reducing cellular cholesterol. Dynasore represents an interesting treatment to reduce cellular cholesterol and will be useful to better understand the complexity of cholesterol trafficking and homeostasis for the development of further strategies to limit the impact of CDCs.

## MATERIALS AND METHODS

### Reagents and inhibitors

Recombinant PLO was purified as previously described (21), and the anti-PLO (*α*-PLO) goat antiserum (21) was a generous gift from Professor B. H. Jost (University of Arizona, Tucson, AZ, USA). Ultrapure LPS was from InvivoGen (Toulouse, France), whereas SLO, DTT, nigericin, staurosporine, and rapamycin were purchased from Sigma-Aldrich (Gillingham, United Kingdom). The ERK1/2 inhibitor peptide (#328000), p38 inhibitor SB203580 (#559398), JNK inhibitor II (#420128), MEK1/2 inhibitor UO126 (#662005), MEK1 inhibitor PD098059 (#513001), and dynamin inhibitor 1 Dynasore (#324410) were all purchased from Calbiochem (Nottingham, United Kingdom). The dynamin inhibitor peptide (1775) was purchased from Tocris (R&D Systems, Minneapolis, MN, USA). The mutant PLO (dsPLO) was a kind gift from Professor M. Palmer (University of Waterloo, Waterloo, ON, Canada) and generated as previously described (22).

### Endometrial stromal cell isolation and cell culture

Isolation and culture of endometrial stromal cells were performed as described previously (15, 23, 24). In brief, bovine uteri were collected at a local abattoir, as part of the routine process of the abattoir, from postpubertal nonpregnant animals with no evidence of genital disease or microbial infection. Stromal cells were isolated following enzymatic digestion of the endometrium and differential adhesion to cell culture plates, with cell purity verified as described previously (24). Stromal cells were maintained in complete culture medium composed of RPMI-1640 (Gibco, Gaithersburg, MD, USA), 10% fetal bovine serum (FBS; Gibco), 50 IU/ml penicillin, 50 *µ*g/ml streptomycin, and 2.5 *µ*g/ml amphotericin B (all from Sigma-Aldrich). HeLa cells were purchased from American Type Culture Collection (Manassas, VA, USA) and grown in DMEM (Gibco) containing 10% FBS, 50 IU/ml penicillin, 50 *µ*g/ml streptomycin, and 2.5 *µ*g/ml amphotericin B. Cells were incubated at 37°C in air with 5% CO_2._

### Western blotting

To examine activation of intracellular signaling pathways by CDCs, endometrial stromal cells were seeded in 6-well tissue culture plates (Techno Plastic Products AG, Trasadingen, Switzerland) (5 × 10^5^/well) and incubated overnight in serum-free medium before treatment with control medium or medium containing 50 hemolytic units (HU) of PLO. After treatment, cells were washed in Dulbecco’s PBS (DPBS; Gibco) and lysed by adding 150 *µ*l of a solution containing RIPA buffer, protease inhibitors, and phosphatase inhibitor cocktail 2 and 3 (all from Sigma-Aldrich). The cell lysate was collected and centrifuged at 8000 × *g* for 10 minutes at 4°C, and the protein concentration was measured by DC Assay (Bio-Rad, Hercules, CA, USA). For protein detection, Western blotting was performed according to standard procedures, as described previously (23, 24). The following primary antibodies were used: rabbit anti-ERK1-2 (#17942; Abcam Incorporated, Cambridge, MA, USA); mouse anti-diphosphorylated ERK1/2 (M8159; Sigma-Aldrich); rabbit anti-MAPK p38*α* (APO3041SU-N; Acris, Herford, Germany); rabbit anti-MAPK p38*α* pThr180/pTyr182 (APO5898PU-N; Acris); rabbit anti-SAPK/JNK (#9252; Cell Signaling Technology, Danvers, MA, USA); rabbit anti-p-SAPK/JNK (#9251; Cell Signaling Technology); rabbit anti *α*-tubulin (#2125; Cell Signaling Technology); rabbit anti-LC3B [(protein light-chain 3B) #2775; Cell Signaling Technology]; and mouse anti-Dynamin (ab14448; Abcam Incorporated). After incubation with the appropriate secondary antibody, immune-reactive bands were visualized by using Clarity Western ECL Substrate (Bio-Rad) and images collected using a Chemi-Doc imaging system (Bio-Rad). Quantification of the bands was done using the ImageJ software (National Institutes of Health, Bethesda, MD, USA), as described previously (25).

### Cell survival assays

To specifically explore the impact of PLO or SLO, 5 × 10^4^/well endometrial stromal cells in 24-well tissue culture plates were incubated overnight in 1 ml serum-free medium and then treated for 1 h with control culture medium or medium containing PLO or SLO using the range of concentrations specified in RESULTS. Cell survival was assessed by the mitochondria-dependent reduction of 3-(4,5-dimethylthiazol-2-yl)-2,5-diphenyltetrazolium bromide (MTT) to formazan, as described previously (26). Briefly, for the MTT assay, once the supernatants were removed, fresh culture medium containing 1 mg/ml MTT was added and incubated with the cells for 1 h; the medium was then removed, and the cells were lysed with DMSO for the measurement of optical density (OD) at 570 nm using a microplate reader (POLARstar Omega; BMG Labtech, Offenburg, Germany), with data expressed as the percentage of control cell survival.

The alamarBlue assay was used to examine the kinetics of cell survival (Pierce, Rockford, IL, USA). AlamarBlue is a tetrazolium-based dye, incorporating resazurin and resorufin as oxidation-reduction indicators that yield colorimetric changes and a fluorescent signal in response to metabolic activity (27). Stromal cells were grown in 24-well plates and treated with 100 HU PLO prior to adding alamarBlue reagent. After 1, 2, 4, and 24 hours incubation at 37°C in air with 5% CO_2_, fluorescence was measured at 545/590 nm (excitation/emission), with data expressed as the percentage of control cell survival.

### Immunofluorescence

To analyze the induction of autophagy, 1 × 10^4^/well stromal cells were cultured overnight in 8-well chamber slides (BD Biosciences, Oxford, United Kingdom) in complete or serum-free medium. The following day, cells were treated for 3 hours with control medium or media containing 5, 10, or 20 HU PLO, or with 100 nM rapamycin (Sigma-Aldrich) or 1 *μ*M nigericin potassium ionophore (Sigma-Aldrich) as a positive control to induce autophagy. After treatment, cells were washed with DPBS and fixed in 4% paraformaldehyde for 30 minutes, the slides then were rehydrated in PBS for 1 h and blocked with a solution containing 1% bovine serum albumin (BSA). Slides were incubated overnight with the rabbit anti-LC3B antibody (#2775), then washed in PBS, after which the secondary Alexa Fluor 594 goat anti-rabbit antibody was added (Molecular Probes, Eugene, OR, USA) and incubated for 1 h at room temperature. The slides were washed and stained with Vectashield containing DAPI (Vector Laboratories, Burlingame, CA, USA) and analyzed by confocal microscopy (LSM; Carl Zeiss, Jena, Germany). For the analysis of nuclear morphology, stromal cells were treated with 100 HU PLO for 1 hour, in the presence of Dynasore or vehicle, and stained with Vectashield mounting medium containing propidium iodide (Vector Laboratories). Morphology of cells was analyzed with an Olympus BX51 light microscope (Olympus, Southend-on-Sea, United Kingdom), and at least 100 cells per treatment, in 3 independent experiments, were scored for the presence of nuclei with apoptotic/necrotic phenotype. For the analysis of lipid rafts, 1 × 10^4^/well endometrial stromal cells or HeLa cells were cultured overnight in 8-well chamber cover slides in serum-free medium. The following day, cells were treated with Dynasore or M*β*CD before control medium or medium containing 100 HU PLO was added. To stain lipid rafts, the cells were incubated with cholera toxin B (CTB)-FITC (Sigma-Aldrich) for 30 minutes before fixing in 4% paraformaldehyde and blocking in 1% BSA. The slides were stained with Vectashield mounting medium containing propidium iodide and analyzed with an Olympus BX51 light microscope.

### Hemolytic activity

To determine the potency of CDCs, the hemolytic activity of PLO and SLO was measured using a standard hemolysis assay, as described previously (15). To determine whether Dynasore protects red blood cells against CDC-mediated hemolysis, a kinetic hemolysis assay was used, also as described previously (22). In brief, horse red blood cells (Oxoid, Basingstoke, United Kingdom) in a 96-well plate at a final concentration of 0.5% were incubated for 30 min in DPBS containing vehicle, 2.5 mM M*β*CD, or 20 *μ*M Dynasore using 4 replicates per treatment, followed by the addition of PLO or SLO. Hemolysis was monitored at 25°C, and the OD_620_ measured every minute for 40 minutes using a microplate reader (POLARstar Omega). To determine whether Dynasore binds directly to CDCs to inhibit cell lysis, vehicle, PLO, or SLO was incubated in DPBS or DPBS containing 20 *μ*M Dynasore for 5 or 30 minutes before the addition of 0.5% horse red blood cells, and measurement of OD_620_ was performed every minute for 40 minutes using 4 replicate wells per treatment. The experiments were repeated on 4 independent occasions.

### ATP and cholesterol measurement

ATP was measured using the ATP Determination Kit (Molecular Probes) based on luciferase’s absolute requirement for ATP in producing light (emission maximum ∼560 nm at pH 7.8). Cellular cholesterol content was measured using the Amplex Red Cholesterol Assay Kit (Invitrogen, Life Technologies, Paisley, United Kingdom), according to the manufacturer’s instructions.

### ELISA

To further explore the role of Dynasore in cell membranes, stromal cells were treated with 100 ng/ml LPS for 24 hours because inflammatory mediator secretion in response to LPS depends on lipid rafts (28). Concentrations of IL-6 and IL-8 in cell culture supernatants were measured by ELISA according to the manufacturer’s instructions. Bovine IL-6 Screening Set ESS0029 (Thermo Fisher Scientific, Waltham, MA, USA) and Human CXCL8/IL-8 DuoSet DY208 (R&D Systems Europe Limited, Abingdon, United Kingdom) were used as previously described (28). The Human CXCL8/IL-8 DuoSet was previously validated for measurement of bovine IL-8 (29).

### Short interfering RNA

Primary endometrial stromal cells were transfected with Lipofectamine RNAiMAX Reagent (Invitrogen) and short interfering RNA (siRNA) (designed using Dharmacon siDESIGN Center; Thermo Fisher Scientific) targeting Dynamin (sense, 5′-GGACAUCGAUGGUAAGAAAUU-3′; antisense, 5′-UUUCUUACCAUCGAUGUCCUU-3′), as described previously (23) In brief, RNAiMAX-RNAi duplex complexes were formed by adding 50 pmol siRNA to 500 *μ*l Opti-MEM I Reduced Serum Media (without antibiotics; Invitrogen) in each well of a 6-well plate. For controls, 50 pmol ON-TARGETplus Non-targeting siRNA #1 (Dharmacon-Thermo Fisher Scientific, Loughborough, United Kingdom) was used instead of the targeted siRNA.

### Statistical analysis

Data are presented as the arithmetic mean (SD) of at least 3 independent experiments. Statistical analyses were performed using GraphPad Prism, version 5 (La Jolla, CA, USA), and data were analyzed using ANOVA with Dunnett’s *post hoc* comparison test. Significance was ascribed at *P* < 0.05.

## RESULTS

### PLO induces activation of the MAPK pathways

**Figure 1.**
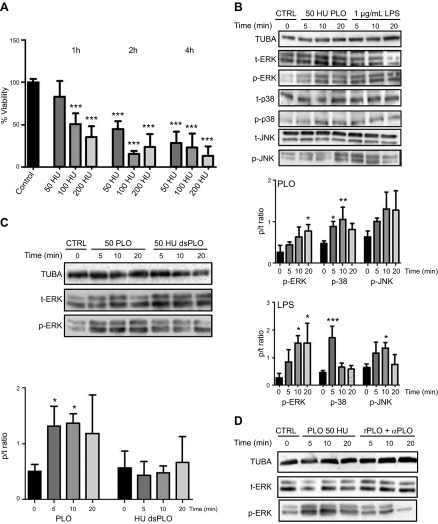
PLO induces phosphorylation of MAPK. *A*) Cytolysis of endometrial stromal cells treated for 1, 2, or 4 hours with the indicated concentration of PLO, as determined by MTT assay. *B*) Western blotting analysis of total (t-) and phosphorylated (p-) ERK1/2, p38, and JNK in endometrial stromal cells treated with 50 HU PLO or 1 *μ*g/ml LPS for the indicated times, with *α*-tubulin (TUBA) used as a loading control (CTRL). *C*) Western blotting analysis of phosphorylation of ERK in endometrial stromal cells treated with 50 HU PLO or 50 HU dsPLO for the indicated times, with *α*-tubulin used as a loading control. Data in graphs are presented as the mean (SD) of 3 independent experiments, and data were analyzed using ANOVA and Dunnett’s *post hoc* test. Values differ from control: *P < 0.05; **P < 0.01; ***P < 0.001. *D*) Western blotting analysis of phosphorylation of ERK in endometrial stromal cells treated with 50 HU PLO, after 1 hour preincubation with medium alone or medium containing *α*-PLO antibody, with *α*-tubulin used as a loading control.

Several members of the CDC family activate MAPK at sublytic CDC concentrations (11, 12, 30). To test if PLO was able to induce a similar activation, primary endometrial stromal cells were treated with a range of concentrations of PLO for 1, 2, and 4 hours to identify a sublytic concentration (**Fig. 1*A***). The experiment was performed in stromal cells in serum-free medium because FBS activates the ERK1/2 pathway (31, 32). According to the MTT data, the LD_50_ was approximately 100 HU after 1 hour; therefore, stromal cells were treated with a sublytic concentration of 50 HU PLO for up to 20 minutes, using LPS as a positive control to induce phosphorylation of MAPK (23) (**Supplemental Fig. 1*A***). Treatment with PLO induced a time-dependent phosphorylation of ERK1/2 and p38, whereas phosphorylation of JNK was less evident [Fig. 1*B*, with quantification in the panel below (*n* = 3 animals), and Supplemental Fig. 1*B*]. To further confirm that the effect on the MAPK pathway was due to the effect of PLO, cells were treated with the dsPLO, which is able to bind to cell membranes but does not form oligomers (22). Indeed, PLO induced more phosphorylation of ERK1/2 compared with dsPLO (Fig. 1*C*, with quantification below; *n* = 3 animals). Moreover, to provide further evidence for the effect of PLO, PLO was incubated with the specific antibody *α*-PLO, prior to treatment of cells, which reduced the phosphorylation of ERK1/2 compared with PLO treatment alone (Fig. 1*D*).

### PLO induces autophagy

**Figure 2.**
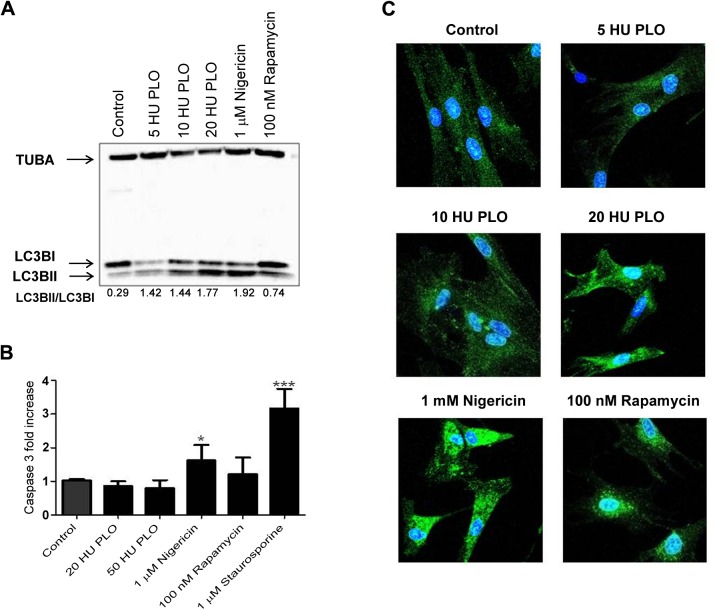
PLO induces activation of autophagy. *A*) Western blotting analysis of LC3BI-to-LC3BII conversion in endometrial stromal cells treated for 3 hours with the indicated concentrations of PLO, or with established inducers of autophagy nigericin and rapamycin, with *α*-tubulin used as a loading control. The numerical ratio of LC3BII:LC3BI is indicated under each treatment, with 1 representative experiment of 3 shown. *B*) Caspase-3 activity in endometrial stromal cells treated with the indicated concentration of PLO or with nigericin, rapamycin, or staurosporine as a positive control. Assay for caspase-3 was performed using the fluorogenic substrate DEVD-AMC, and data are reported as a fold of control. Data are presented as the mean (SD) of 3 independent experiments and analyzed using ANOVA and Dunnett’s *post hoc* test. Values differ from control: *P < 0.05; ***P < 0.001. *C*) Immunocytochemistry analysis of LC3B induction in endometrial stromal cells treated for 3 h with the indicated concentrations of PLO, rapamycin, or nigericin. DNA is stained blue; LC3B is stained green.

Autophagy is an intracellular catabolic process activated in stressed cells (33). The autophagic pathway also assists the elimination of certain intracellular pathogens (34). Furthermore, autophagy is induced in response to some pore-forming toxins (3). To evaluate if PLO induces autophagy, endometrial stromal cells were treated with 5, 10, or 20 HU PLO, using 2 common activators of autophagy (nigericin and rapamycin) as positive controls (35, 36). After 3 hours, cells were collected, and Western blotting was performed to detect the conversion of the LC3-I form into the processed LC3-II, a marker of autophagy activation (37). A PLO concentration-dependent increase in LC3-II was observed (**Fig. 2*A***) in the absence of apoptosis, as determined by the lack of caspase-3 activation (Fig. 2*B*). Moreover, treatment with PLO induced a punctate distribution of LC3 in cells, similar to that induced by rapamycin and nigericin (Fig. 2*C*), providing further evidence that PLO induces autophagy.

### Dynasore protects against PLO-induced cytolysis

**Figure 3.**
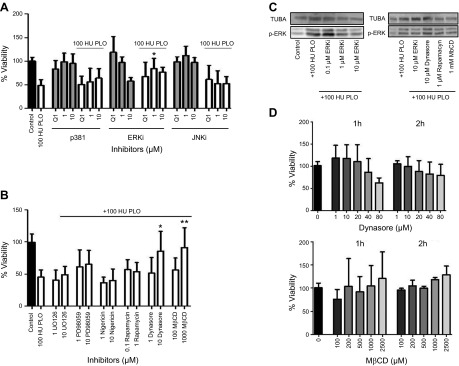
Inhibitor screening of PLO-treated stromal cells. *A*) Viability of endometrial stromal cells pretreated for 30 minutes with the indicated concentrations of MAPK inhibitors and then treated with control media or media containing 100 HU PLO. Viability was assessed by MTT assay, and data are presented as the percentage of control cell survival. *B*) Cytolysis of endometrial stromal cells pretreated for 30 min with the indicated concentrations of inhibitors and then treated with 100 HU PLO. Cell survival was assessed by MTT assay, and data are presented as the percentage of control cell survival. Data in (*A*) and (*B*) are presented as the mean (SD) of 5 independent experiments and were analyzed using ANOVA and Dunnett’s *post hoc* test. Values differ from 100 HU PLO treatment: *P < 0.05; **P < 0.01. *C*) Western blotting analysis of phosphorylation of ERK in endometrial stromal cells treated with 100 HU PLO, in the presence of the indicated inhibitors, with *α*-tubulin used as a loading control. *D*) alamarBlue assay of endometrial stromal cells treated with the indicated concentration of Dynasore or M*β*CD for 1 and 24 hours. Data are presented as the mean (SD) of 4 independent experiments.

To evaluate the functional effect of activation of the MAPK pathway, stromal cells were pretreated for 30 minutes with the specific inhibitor of p38 MAPK SB203580, the ERK1/2 inhibitor peptide, and the JNK inhibitor II, using 3 different concentrations (0.1, 1, and 10 *μ*M), before challenging cells with a lytic dose of 100 HU PLO for 1 h. Although the p38 and the JNK inhibitors had no significant effects (**Fig. 3*A***), surprisingly, inhibition of ERK conferred endometrial stromal cells with some protection against PLO, which is the opposite effect for other cells and CDCs (3, 38, 39). The protective effect observed with ERK inhibition might be explained by the reported proapoptotic role of ERK (40, 41). Thus, to further analyze the prosurvival mechanism, the MEK1/2 inhibitor UO126 and the MEK1 inhibitor PD098059 were tested, as well as the autophagy inducers rapamycin and nigericin, the dynamin inhibitor I Dynasore, and the cyclodextrin M*β*CD. The last 2 compounds affect clathrin-dependent endocytosis (42) and cholesterol homeostasis, respectively (15, 17). Although MEK inhibitors and autophagy inducers had limited effects on endometrial stromal cell survival when treated with PLO, Dynasore protected against cytolysis similar to M*β*CD (Fig. 3*B*), which protects against PLO by reducing stromal cell cholesterol content (15). Dynasore acts as a potent inhibitor of clathrin-endocytic pathways that depend on dynamin, by blocking coated vesicle formation within seconds (43, 44). This protective role of Dynasore was not linked with reduced phosphorylation of ERK, as demonstrated by Western blotting analysis (Fig. 3*C*).

**Figure 4.**
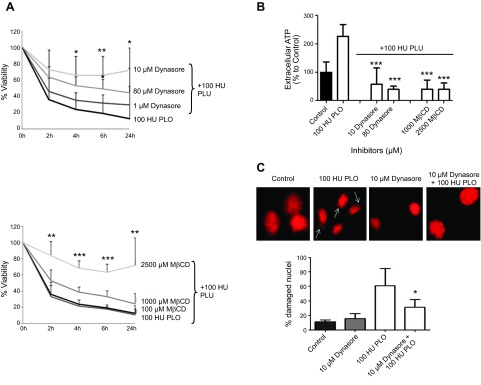
Dynasore confers protection against PLO. *A*) Viability of endometrial stromal cells pretreated for 30 minutes with the indicated concentrations of Dynasore or M*β*CD and then treated with 100 HU PLO. Cell viability was assessed by alamarBlue assay, and data are presented as the percentage of control cell survival. Data are presented as the mean (SD) of 4 independent experiments and were analyzed using ANOVA and Dunnett’s *post hoc* test. Values differ from 100 HU PLO treatment: *P < 0.05; **P < 0.01; ***P < 0.001. *B*) Measurement of extracellular ATP, as determined by a luciferase assay, in endometrial stromal cells pretreated with the indicated concentration of Dynasore or M*β*CD, and then treated with 100 HU PLO. Data are presented as the mean (SD) of 4 independent experiments and analyzed by ANOVA with Dunnett’s *post hoc* test. Values differ from 100 HU PLO treatment: ***P < 0.001. *C*) Fluorescence analysis of endometrial stromal cells pretreated with 10 *μ*M Dynasore and then treated with 100 HU PLO. At least 100 cells per treatment were scored for the presence of nuclei with apoptotic or necrotic phenotype in 3 independent experiments. Data were analyzed by ANOVA and Dunnett’s *post hoc* test. Values differ from 100 HU PLO treatment: *P < 0.05.

To further characterize the effect of Dynasore and M*β*CD on endometrial stromal cells, we first evaluated potentially toxic effects of these inhibitors using concentrations ranging from 1 to 80 *μ*M for Dynasore and 100 *μ*M to 2.5 mM for M*β*CD. As reported in Fig. 3*D*, AlamarBlue assay showed that only concentrations around 80 *μ*M Dynasore affected cell viability, whereas no toxic effects were shown by M*β*CD, even at the highest concentration. Both Dynasore and M*β*CD conferred resistance to 100 HU PLO for 24 h as demonstrated by alamarBlue assay (**Fig. 4*A***), using 3 concentrations of Dynasore (1, 10, and 80 *μ*M) and 3 of M*β*CD (100 *μ*M and 1 and 2.5 mM). To provide further evidence that both inhibitors are effective in protecting endometrial stromal cells against PLO-induced cytolysis, we investigated the ability to prevent the common effect of pore-forming toxins on target cells in inducing membrane permeabilization and rapid ATP depletion (45). Measurement of ATP showed that PLO treatment induces an increase of ATP in the supernatant twice the level of the control, but pretreatment with Dynasore or M*β*CD inhibited ATP depletion from the cells (Fig. 4*B*). Moreover, propidium iodide staining revealed that pretreatment with Dynasore reduced the number of cells with damaged nuclei (Fig. 4*C*), supporting the concept that Dynasore decreased the cellular sensitivity to PLO.

### Dynasore acts on cholesterol content *via* disruption of lipid rafts

**Figure 5.**
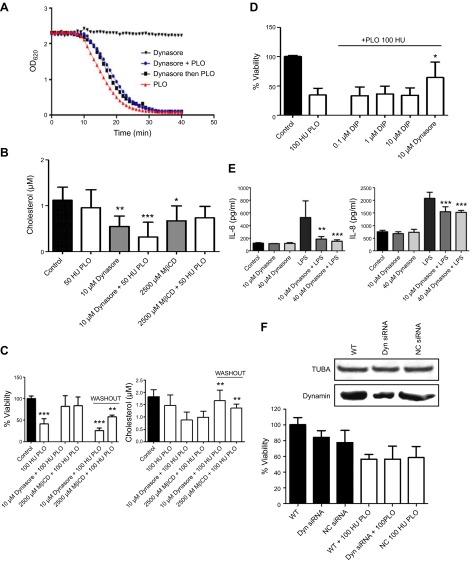
Dynasore acts on cholesterol. *A*) Horse red blood cells were treated with 10 *μ*M Dynasore, Dynasore mixed with PLO, PLO alone, or red blood cells were incubated with Dynasore for 30 minutes and then PLO applied; OD_620_ was measured every minute. Data are presented as the mean of 4 replicates for each treatment, and the experiment is typical of 3 independent experiments. *B*) Cholesterol measurement in stromal cells pretreated for 30 minutes with Dynasore or M*β*CD and then treated with control medium or medium containing 50 HU PLO with cholesterol measured using the Amplex Red Cholesterol Assay Kit. Data are presented as the mean (SD) of 6 independent experiments and analyzed by ANOVA with Dunnett’s *post hoc* test. Values differ from control: *P < 0.05; **P < 0.01; ***P < 0.001. *C*) Viability (left panel) and cholesterol content (right panel) of endometrial stromal cells pretreated for 30 minutes with Dynasore or M*β*CD before a washout was performed for half-plate and 100 HU PLO was added. Cell survival was assessed by MTT assay, and data are presented as the mean (SD) of 4 independent experiments, with data analyzed by ANOVA with Dunnett’s *post hoc* test. Values differ from control: **P < 0.01; ***P < 0.001. *D*) Viability of endometrial stromal cells pretreated for 30 minutes with Dynasore or dynamin inhibitor peptide (DIP) before 100 HU PLO was added. Cell viability was assessed by MTT assay, and data are presented as the mean (SD) of 4 independent experiments, with data analyzed by ANOVA with Dunnett’s *post hoc* test. Values differ from 100 HU PLO treatment: *P < 0.05. *E*) Concentrations of IL-6 and IL-8 in the supernatant of stromal cells treated for 30 minutes with control medium or medium containing 10 or 40 *μ*M Dynasore and then treated with control medium or medium containing 100 ng/ml LPS for 24 hours. Data are presented as the mean (SD) of 4 independent experiments and analyzed by ANOVA with Dunnett’s *post hoc* test. Values differ from LPS: ***P < 0.001. *F*) Viability of endometrial stromal cells treated for 48 h with Dynamin siRNA (Dyn siRNA) and with nontargeting siRNA (NC siRNA) and therefore treated with 100 HU PLO for 1 hour. Cell viability was assessed by MTT assay, and data are presented as the mean (SD) of 3 independent experiments, with data analyzed by ANOVA with Dunnett’s *post hoc* test. Values differ from wild-type (WT) 100 HU PLO treatment. The efficiency of knockdown was assessed by Western blotting analysis.

To exclude the possibility that the protection conferred by Dynasore was related to binding between Dynasore and PLO, we performed a standard kinetic hemolysis assay where horse red blood cells were treated with Dynasore alone, Dynasore and PLO at the same time, or Dynasore followed after 30 minutes by PLO. As shown in **Fig. 5*A***, Dynasore did not inhibit the action of PLO by direct disruption of the CDC or by protecting the red blood cells. However, as expected, M*β*CD protected red blood cells from PLO-induced hemolysis (**Supplemental Fig. 2*A***).

**Figure 6.**
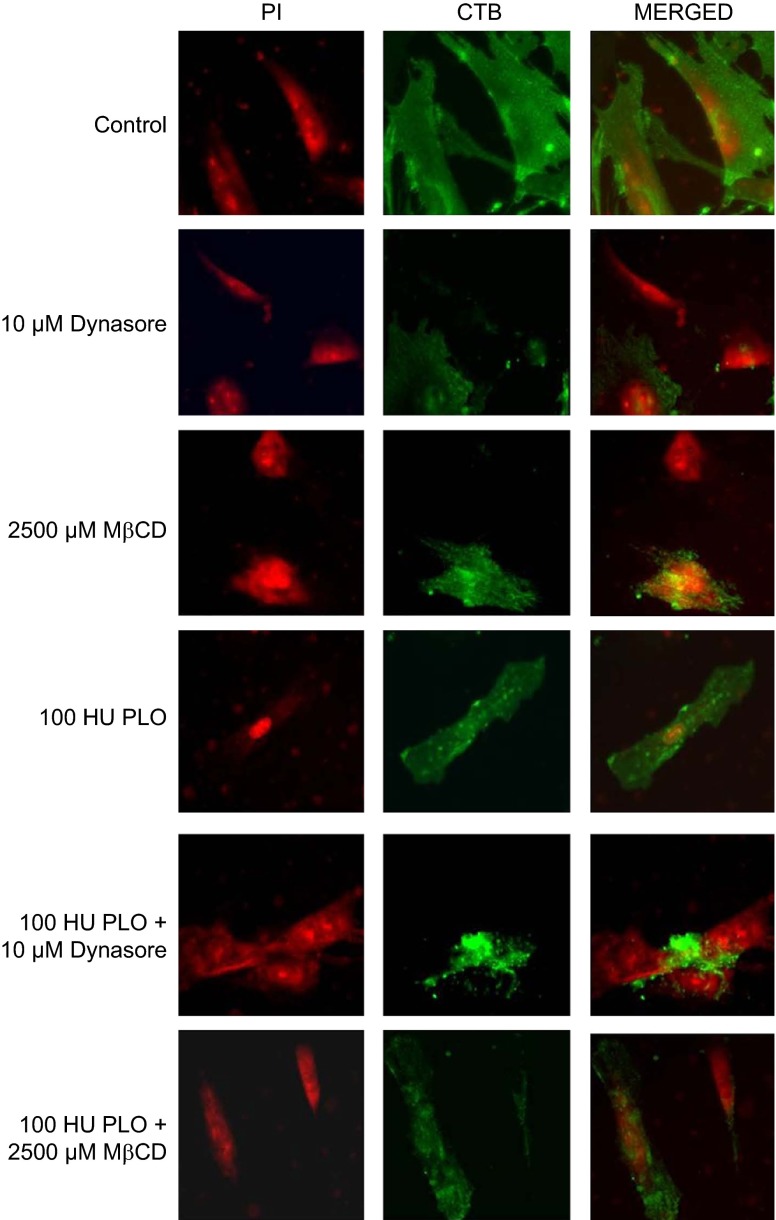
Dynasore and M*β*CD target lipid rafts. Lipid raft staining is shown in endometrial stromal cells pretreated for 30 minutes with 10 *μ*M Dynasore or 2500 *μ*M M*β*CD and then treated with control medium or medium containing 100 HU PLO for 1 hour. Lipid rafts are stained using the CTB-FITC conjugate. DNA stained red; CTB stained green. One representative experiment out of 3 is shown.

Dynasore impairs cholesterol trafficking and transcription of sterol-sensitive genes by promoting accumulation of LDL and free cholesterol within the endolysosomal network (16, 17). Because cholesterol-enriched lipid domains are necessary for the invagination of clathrin-coated pits (42, 46, 47), we hypothesized that Dynasore reduces cellular cholesterol *via* destruction of lipid rafts, to protect endometrial stromal cells against PLO cholesterol binding and the following formation of the pore. Control cells had a cholesterol concentration around 1.2 *μ*M, and treatment with the sublytic dose of 50 HU PLO did not significantly change cholesterol concentration. However, Dynasore and M*β*CD decreased cellular cholesterol concentration in the presence or absence of PLO (Fig. 5*B*). Washout experiments revealed that the effects of Dynasore and M*β*CD were reversible because removal of the inhibitors restores sensitivity to PLO and cholesterol levels (Fig. 5*C* and Supplemental Fig. 3) The dynamin inhibitor peptide was unable to rescue cells from PLO-induced cell death, which implies that the effect of Dynasore was related more to targeting lipid rafts than the GTPase activity of dynamin (Fig. 5*D*). Further evidence that Dynasore targets lipid rafts was reduced production of IL-6 and to a lesser extent IL-8, when stromal cells were pretreated with Dynasore before stimulation with LPS (Fig. 5*E*). Short interference RNA experiments showed that dynamin knockdown does not confer protection against PLO, confirming that the effect of Dynasore occurs in a dynamin-independent way (Fig. 5*F*). To seek more direct evidence that Dynasore targets lipid rafts, we performed immunocytochemical studies using the lipid raft marker, CTB. Treatment with Dynasore or M*β*CD reduced the staining by CTB, both in control and PLO-treated stromal cells, implying that the decrease in cholesterol content occurs *via* disruption of lipid rafts (**Fig. 6**).

### Protective effect of Dynasore against CDCs is a generalized mechanism

**Figure 7.**
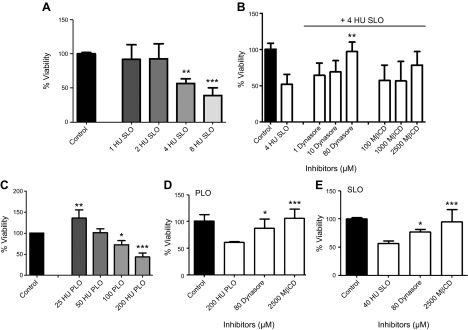
Protective effect of Dynasore against CDCs is a generalized mechanism. *A*) Viability of endometrial stromal cells treated for 1 hour with the indicated concentrations of SLO. Cell survival was assessed by MTT assay. Data are presented as the mean (SD) of 4 independent experiments and were analyzed using ANOVA with Dunnett’s *post hoc* test. Values differ from control: **P < 0.01; ***P < 0.001. *B*) Viability of endometrial stromal cells pretreated for 30 minutes with the indicated concentrations of Dynasore or M*β*CD and then treated with 4 HU SLO. Data are presented as the mean (SD) of 4 independent experiments and were analyzed using ANOVA with Dunnett’s *post hoc* test. Values differ from 4 HU SLO treatment: **P < 0.01. *C*) Viability of HeLa cells treated for 1 hour with the indicated concentrations of PLO. Cell viability was assessed by MTT assay. Data are presented as the mean (SD) of 4 independent experiments and were analyzed using ANOVA with Dunnett’s *post hoc* test. Values differ from control: *P < 0.05; **P < 0.01; ***P < 0.001. *D*) Viability of HeLa cells pretreated for 2 hours with the indicated concentrations of Dynasore or M*β*CD and then treated with 200 HU PLO. *E*) Viability of HeLa cells pretreated for 2 hours with the indicated concentrations of Dynasore or M*β*CD and then treated with 4 HU SLO. Data in (*D*) and (*E*) are presented as the mean (SD) of 4 independent experiments and were analyzed using ANOVA with Dunnett’s *post hoc* test. Values differ from 200 HU PLO or 4 HU SLO treatment: *P < 0.05; ***P < 0.001.

To test if Dynasore influences endometrial cell sensitivity to other members of the CDC family, endometrial stromal cells were treated with SLO from *Streptococcus pyogenes*, which is not a pathogen of the bovine uterus (9). Following DTT activation, necessary for all CDCs except PLO and intermedilysin, stromal cells were treated with a range of concentrations of SLO for 1 hour, and the LD_50_ was 4 HU (**Fig. 7*A***). When endometrial stromal cells were pretreated with Dynasore or M*β*CD, before adding 4 HU SLO, increased cell survival was evident (Fig. 7*B*). A kinetic hemolysis assay was also used to confirm that protection of cells was not related to Dynasore binding to SLO (Supplemental Fig. 2*B*).

**Figure 8.**
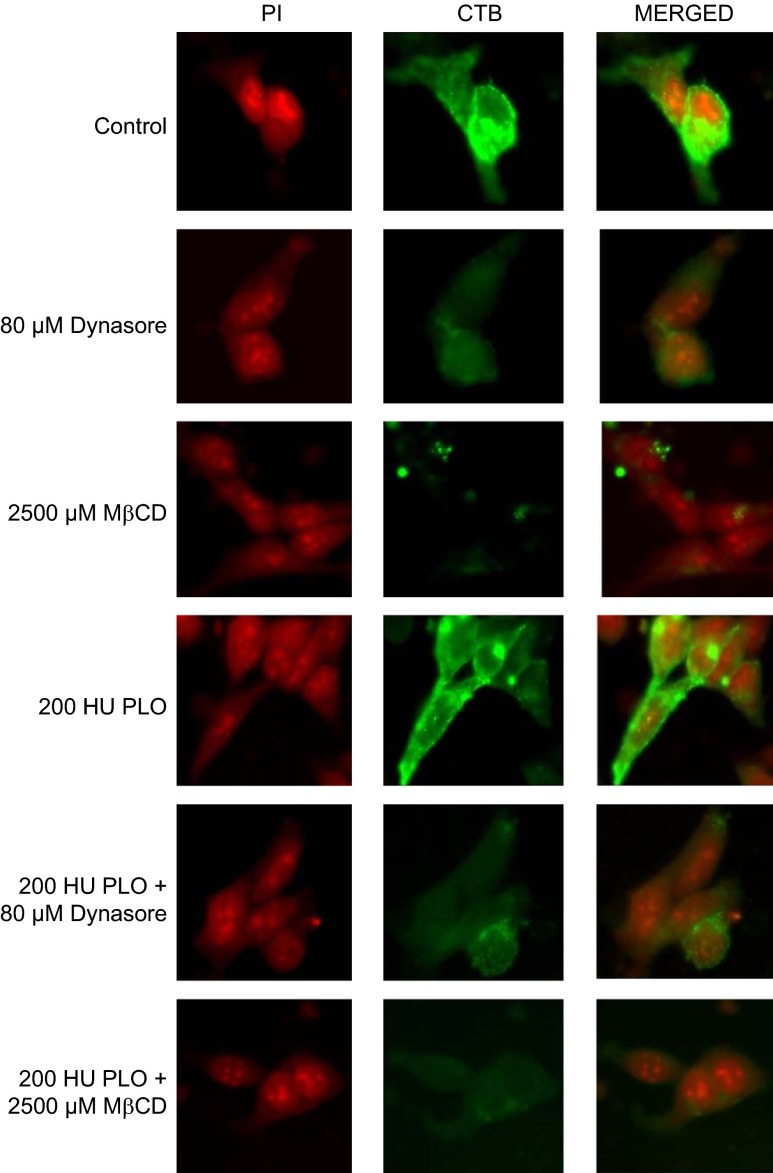
Dynasore and M*β*CD target lipid rafts in HeLa cells. Lipid raft staining is shown in Hela cells pretreated for 2 hours with 80 *μ*M Dynasore or 2500 *μ*M M*β*CD and then treated with control medium or medium containing 200 HU PLO for 1 h. Lipid rafts are stained using the CTB-FITC conjugate. DNA stained red; CTB stained green. One representative experiment out of 3 is shown.

To test if Dynasore protection against CDCs could be applied to other different types than stromal cells, HeLa cells were treated with a range of concentrations of PLO or SLO in the presence of Dynasore or M*β*CD. First, we determined that HeLa cells are sensitive to PLO, as shown in Fig. 7*C*, whereas sensitivity to SLO was already reported (48). Although HeLa cells needed a longer incubation time (2 h *versus* 30 minutes) and a higher concentration (80 *μ*M) of Dynasore compared with endometrial stromal cells, a protective effect of Dynasore was observed with HeLa cells both for PLO (Fig. 7*D*) and SLO (Fig. 7*E*). Fluorescence analysis of HeLa cells showed decreased staining for CTB in Dynasore- and M*β*CD-treated cells, implying that the action on lipid rafts is a general mechanism (**Fig. 8**).

## DISCUSSION

**Figure 9.**
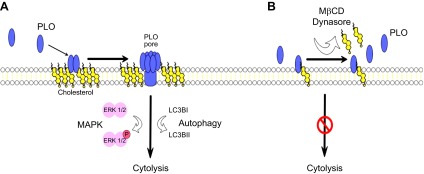
Cellular response to CDCs and protection by Dynasore. *A*) PLO pore formation is dependent on the presence of membrane cholesterol. Cholesterol binding initiates significant secondary and tertiary structural changes in CDCs, which lead to the assembly of a large membrane-embedded *β*-barrel pore complex. Sublytic concentrations of PLO activated the MAPK and autophagy pathways, but these were unable to protect stromal cells against higher concentrations of toxins. *B*) Dynamin GTPase inhibitor Dynasore as well as M*β*CD protected against stromal cytolysis caused by decreasing cellular cholesterol concentration.

The aim of the present study was to examine cellular responses to sublytic concentrations of PLO. This CDC has a unique advantage for *in vitro* studies because PLO is spontaneously active *in vitro*, whereas other CDCs require thiol activation (8). Because *T. pyogenes* commonly causes endometritis (9), primary bovine endometrial stromal cells were used to determine cellular response to PLO, particularly because these stromal cells are highly sensitive to PLO (15). Because many pathways are activated by pore-forming toxins (1, 39), we focused on 3 major areas: phosphorylation of MAPK, autophagy, and cellular cholesterol. Sublytic concentrations of PLO activated the MAPK and autophagy pathways in stromal cells. However, the most striking observation was that the dynamin GTPase inhibitor Dynasore was protective against stromal cytolysis caused by PLO, and this effect was evident for SLO and for HeLa cells. The mechanism of action of Dynasore appears to be to reduce cellular cholesterol content, and Dynasore was as effective as M*β*CD for protection of cells against CDCs (**Fig. 9**).

Sublytic concentrations of PLO induced phosphorylation of p38, ERK, and JNK in stromal cells, as well as induction of the autophagy machinery, as determined by conversion of LC3BI to LC3BII. However, no substantial changes in cell survival were observed when specific inhibitors or activators of these pathways were tested against lytic concentration of PLO, suggesting that increased stromal cell survival was achievable only when cells were incubated with cholesterol-targeting compounds. The protective effect of the M*β*CD against PLO, and to a lesser extent other cyclodextrins, has been noted previously (15). However, a protective role for Dynasore against CDCs has not been reported. The protective effect of Dynasore was also observed using a different toxin (SLO) and the stable HeLa cell line, suggesting that modification of cellular cholesterol content with Dynasore is a valid approach to protect against the effect of bacterial CDCs.

Dynasore acts as a reversible inhibitor of the large GTPase dynamin, which is essential for clathrin-dependent coated vesicle formation (43). Cholesterol-enriched lipid domains are necessary for the invagination of clathrin-coated pits (46, 47). Thus, we hypothesized that Dynasore may also influence the binding of PLO to cholesterol. Indeed, PLO and many other CDCs bind and even form pore-like structures on cholesterol crystals (49), and cholesterol is the only membrane requirement for all but 2 CDCs (intermedilysin and vaginolysin) that require CD59 as well (2, 50, 51). Many studies support a model where CDC monomers preferentially bind to lipid rafts, which are microdomains of the plasma membrane enriched for cholesterol and signaling molecules (52, 53). The lipid raft model is the result of intensive studies on the nature of lipid-sterol interactions using a variety of physical methods (54–56), and nowadays, 2 major observations suggest that cholesterol-lipid interactions play an important role in the formation of rafts in animal cell membranes. First, raft fractions isolated from mammalian cells are found to be enriched in cholesterol (57). Second, disruption of cell membrane-associated cholesterol can induce major changes in the distribution of raft-associated membrane components (58). Targeting of these microdomains represents, therefore, a valid strategy to improve the resistance of eukaryotic cells to CDCs.

Analysis of cholesterol content and immunofluorescence using CTB confirmed that Dynasore as well as M*β*CD decrease the total cellular cholesterol, *vi*a disruption of lipid rafts. This effect was reversible because the levels of cholesterol after washout experiments were restored with both inhibitors but in higher levels with Dynasore than with M*β*CD.

In addition, Dynasore did not bind directly to CDCs because coincubation of Dynasore and PLO or SLO did not affect the kinetics of hemolysis. Presumably the lack of cellular machinery renders ineffective the protective action of Dynasore for red blood cells compared with nucleated stromal cell. Dynamin 1 as well as Dynamin 2 are highly conserved in all vertebrates, including humans and cattle. The dynamin inhibitor peptide competitively blocks binding of dynamin to amphiphysin, thus preventing endocytosis (59). However, pretreatment with this inhibitor and Dynamin-knockdown experiments failed to protect endometrial stromal cells from PLO-induced cell death, supporting the concept that the effect of Dynasore is mainly related to destruction of lipid rafts rather than to inhibition of dynamin. Further functional evidence for this concept comes from the observation in the present study that treatment with Dynasore blocks LPS-induced inflammatory responses in stromal cells because lipid rafts facilitate the LPS response (28, 60). Dynasore could also influence cholesterol membrane content *via* alteration of cholesterol trafficking because previous studies indicated that dynamin inactivation led to the accumulation of free cholesterol within the late endosomal and lysosomal compartments (16).

In conclusion, the present study found that Dynasore provides rapid and reversible protection of cells against PLO and SLO. There was evidence that Dynasore acted to reduce cellular cholesterol and disrupted lipid rafts. We suggest that modification of cellular cholesterol content with Dynasore protects cells against the effects of CDCs.

## Supplementary Material

Supplemental Data

## Abbreviations

BSA: bovine serum albumin
CDC: cholesterol-dependent cytolysin
CTB: cholera toxin B
DPBS: Dulbecco’s PBS
dsPLO: mutant pyolysin
Dynasore: 3-hydroxynaphthalene-2-carboxylic acid-(3,4-dihydroxybenzylidene)-hydrazide
GTPase: guanosine 5′-triphosphatase
HU: hemolytic unit
LC3B: protein light-chain 3B
M*β*CD: methyl-*β*-cyclodextrin
MTT: 3-(4,5-dimethylthiazol-2-yl)-2,5-diphenyltetrazolium bromide
OD: optical density
PI: propidium iodide
PLO: pyolysin
siRNA: short interfering RNA
SLO: streptolysin O
