# Supplementary material for: Protective role of the dynamin inhibitor Dynasore against the cholesterol-dependent cytolysin of Trueperella pyogenes
Source: FASEB J. 2014 Dec 30;29(4):1516–28. doi: 10.1096/fj.14-265207 (PMC4396600; doi:10.1096/fj.14-265207)
Supplement: Supplemental Data [file supp_29_4_1516__index.html]

Protective role of the dynamin inhibitor Dynasore against the cholesterol-dependent cytolysin of Trueperella pyogenes — Protective role of the dynamin inhibitor Dynasore against the cholesterol-dependent cytolysin of Trueperella pyogenes — Supplemental Data 

# Protective role of the dynamin inhibitor Dynasore against the cholesterol-dependent cytolysin of *Trueperella pyogenes*

## Supplemental Data

**Files in this Data Supplement:**

- Supplemental Data
- Supplemental Data
- Supplemental Data
- Supplemental Data
