# Supplementary material for: Protective role of the dynamin inhibitor Dynasore against the cholesterol-dependent cytolysin of Trueperella pyogenes
Source: FASEB J. 2014 Dec 30;29(4):1516–28. doi: 10.1096/fj.14-265207 (PMC4396600; doi:10.1096/fj.14-265207)
Supplement: Supplemental Data [file supp_fj.14-265207_Supplemental_Figures.pdf]

Supplemental Information

Suppl. Figure 1. Pyolysin induces activation of MAPK pathway. (A) Endometrial stromal cells were treated for 20 min with the indicated concentration of LPS and after cells were lysed for Western blotting analysis to detect phosphorylation of ERK. was used as loading control. (B) Western blotting analysis of MAP kinases activation in endometrial stromal cells treated with 50 HU PLO or LPS (1 mg/mL) for the indicated time points in three independent experiments. α–tubulin is used a loading control.

Suppl. Figure 2. Effect of Dynasore and MβCD on haemolysis caused by CDCs. (A) Horse red blood cells were incubated for 30 min at 37 C in media alone or medium containing the indicated concentrations of MβCD or Dynasore, and then treated with 50 HU PLO. OD_{620} was measured every minute. Data are presented as the mean of 4 replicates for each treatment, and the experiment is typical of 4 independent experiments. (B) Horse red blood cells were treated with 10 μM Dynasore, Dynasore mixed with SLO, SLO alone, or red blood cells were incubated with Dynasore for 30 min and then SLO was applied; OD_{620} was measured every minute. Data are presented as the mean of 4 replicates for each treatment, and the experiment is typical of 3 independent experiments.

Suppl. Figure 3. Effect of Dynasore and MβCD washout on lipid rafts staining. Lipid rafts staining in HeLa cells pre-treated for 2h with 80 μM Dynasore or 2500 μM MβCD before a washout in DMEM was performed in half of chamber slides for a supplementary 30 min. Lipid rafts are stained using the CTB-FITC-conjugated. DNA stained red, CTB stained green. One representative experiment out of three is shown.
