# Supplementary figures and images for: Protective role of the dynamin inhibitor Dynasore against the cholesterol-dependent cytolysin of Trueperella pyogenes
Source: FASEB J. 2014 Dec 30;29(4):1516–28. doi: 10.1096/fj.14-265207 (PMC4396600; doi:10.1096/fj.14-265207)

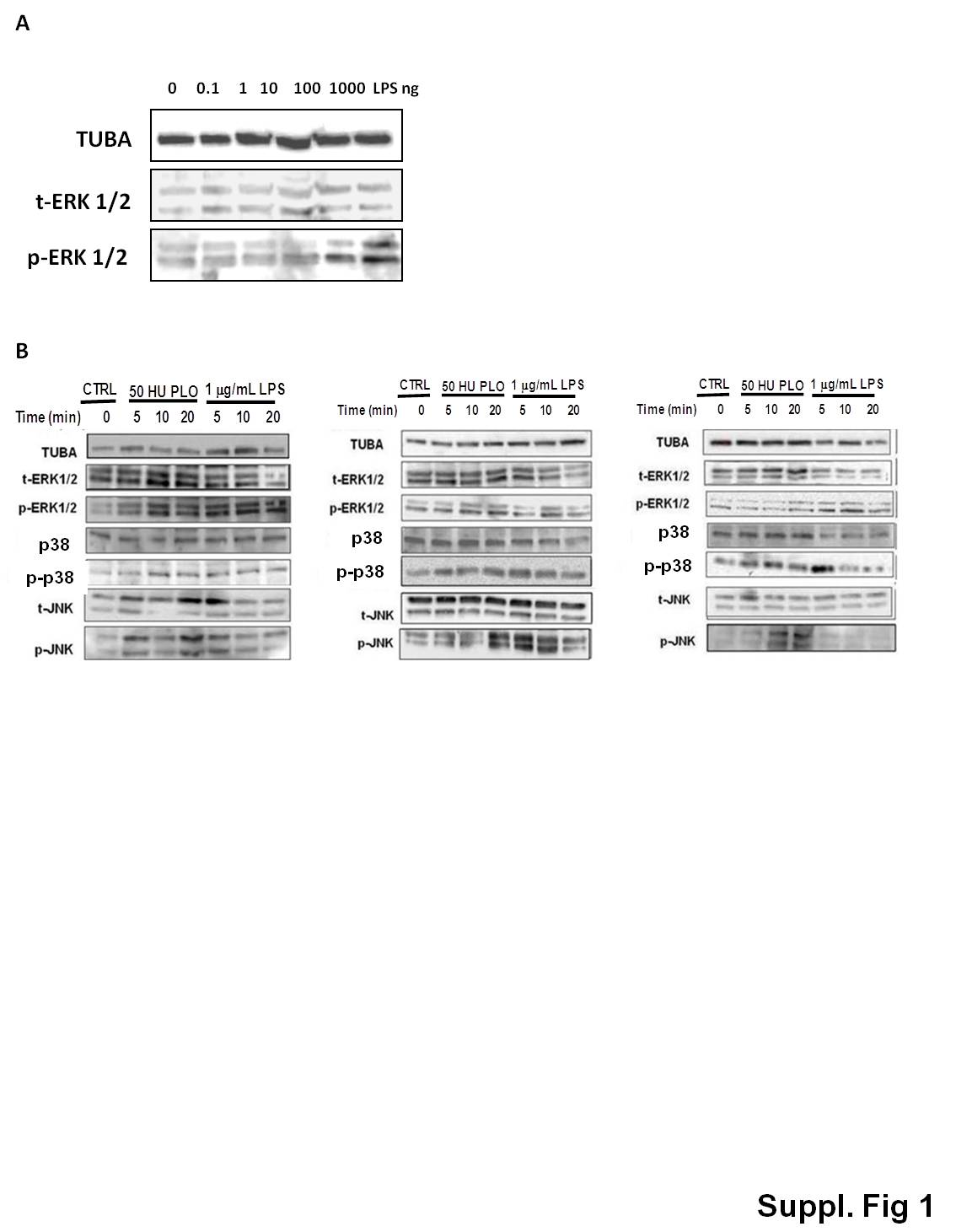

Supplement: Supplemental Data [file supp_fj.14-265207_Supplemental_Figure1.tif]

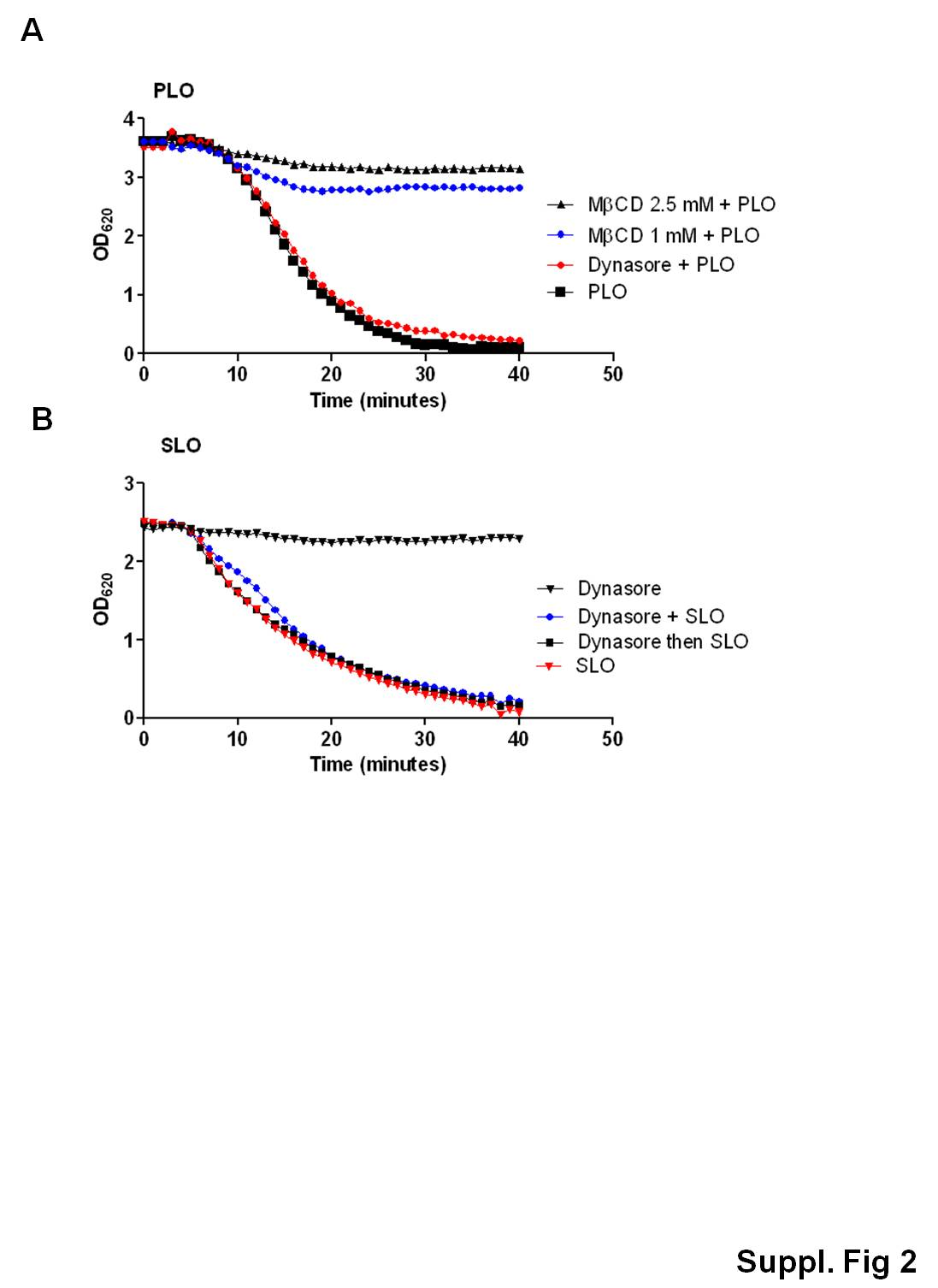

Supplement: Supplemental Data [file supp_fj.14-265207_Supplemental_Figure2.tif]

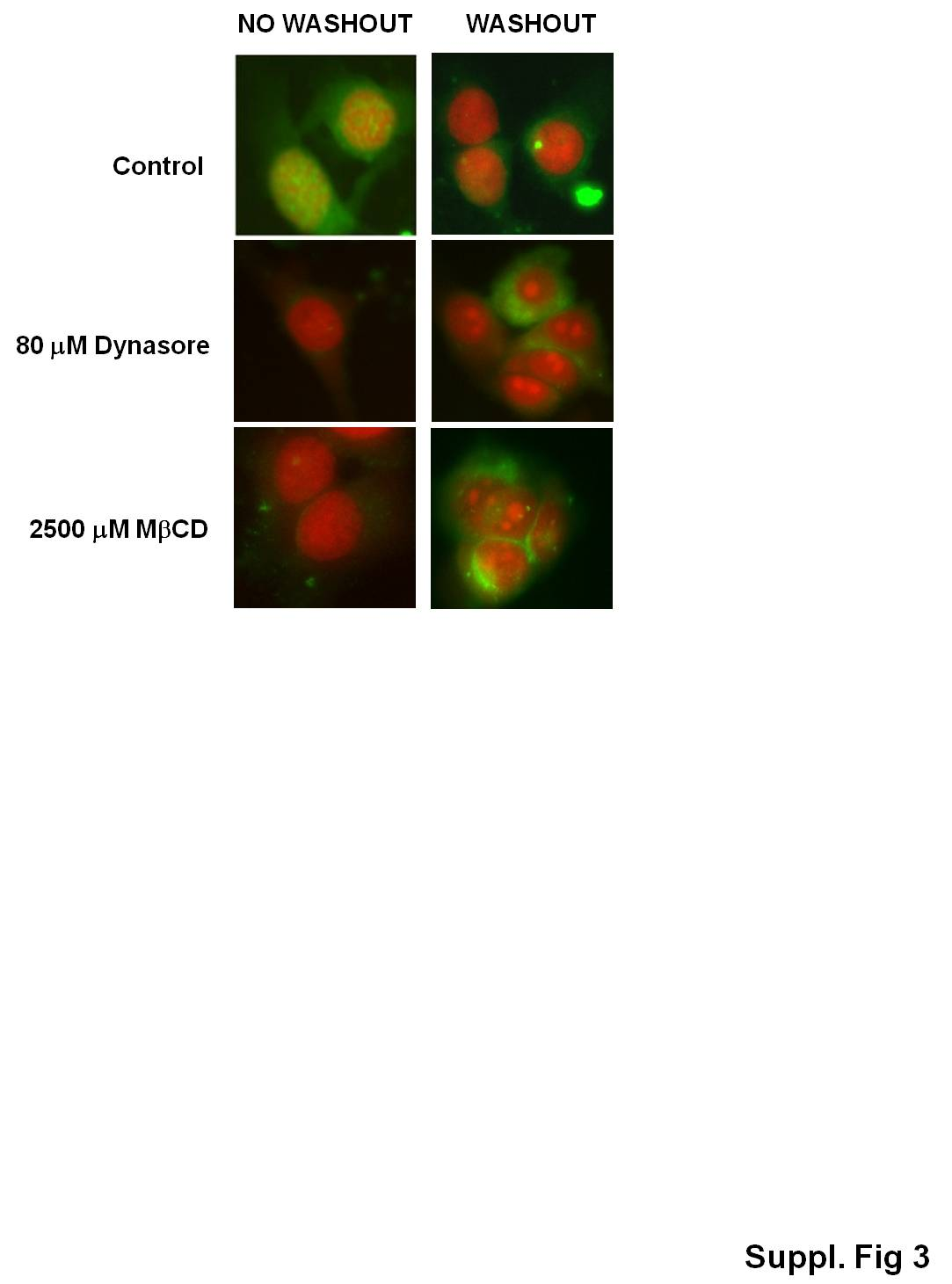

Supplement: Supplemental Data [file supp_fj.14-265207_Supplemental_Figure3.tif]
